# Sustainable adhesive from waste expanded polystyrene: Performance governed by solvent-substrate interplay

**DOI:** 10.1038/s41598-026-42596-8

**Published:** 2026-04-03

**Authors:** Rama AL Jobarani, Hassan Alkurdi, Fawaz Deri

**Affiliations:** 1 Department of Chemistry, Faculty of science, Materials Rheology Laboratory, Damascus University, Damascus, Syria; 2 Department of Chemistry, Faculty of Sciences, Damascus University, Damascus, Syria; 3 Department of Chemistry, Damascus University, Damascus, Syria

**Keywords:** Chemistry, Engineering, Materials science

## Abstract

This study presents a sustainable approach for converting post‑consumer Expanded Polystyrene (EPS) into a high‑performance adhesive. EPS was dissolved in various organic solvents and modified with PMMA, and the resulting formulations were evaluated chemically, Rheologically, and mechanically. Solubility trends correlated with Hansen Solubility Parameters, with toluene showing the highest dissolution efficiency (RED = 0.65). Rheological analysis revealed near‑Newtonian to mildly shear‑thinning behavior (STI = 0.018–0.203), indicating viscosity stability suitable for application. Mechanical testing demonstrated strong substrate‑dependent performance: xylene‑based adhesives achieved high bonding strength on non‑polar leather (≈ 778 KPa), while MEK‑based formulations exhibited superior adhesion to wood (≈ 755 KPa) due to enhanced hydrogen bonding. Incorporation of PMMA further reinforced the adhesive network, yielding the highest tensile strength (≈ 1969 KPa). All formulations bonded effectively to polyurethane and showed robust qualitative adhesion to ceramic. These findings validate the feasibility of upcycling EPS waste into a versatile, high‑strength adhesive, offering a practical and environmentally beneficial solution for plastic waste management.

## Introduction

 Expanded polystyrene (EPS), commercially known as thermocol, is a lightweight, rigid polymer extensively used in packaging, insulation, and disposable consumer products. Global production exceeds 15 million metric tons annually; however, end-of-life management remains problematic due to inadequate recycling infrastructure and limited reuse pathways. In the United States alone, over 2.3 million tons of EPS waste are landfilled yearly, with recycling rates below 2%^1,2^.

A key environmental challenge posed by EPS is its extremely low bulk density, which leads to disproportionate spatial occupation in waste systems. Experimental measurement of EPS samples in this study revealed that one cubic meter weighs only 1440 g, corresponding to a bulk density of 1.44 kg/m³. This volumetric inefficiency means that minimal mass occupies substantial landfill volume, complicating logistics related to collection, transport, and disposal. Moreover, the low mass and high surface area of EPS make it prone to wind dispersal, resulting in widespread littering in terrestrial and aquatic environments.

**Fig. 1:**
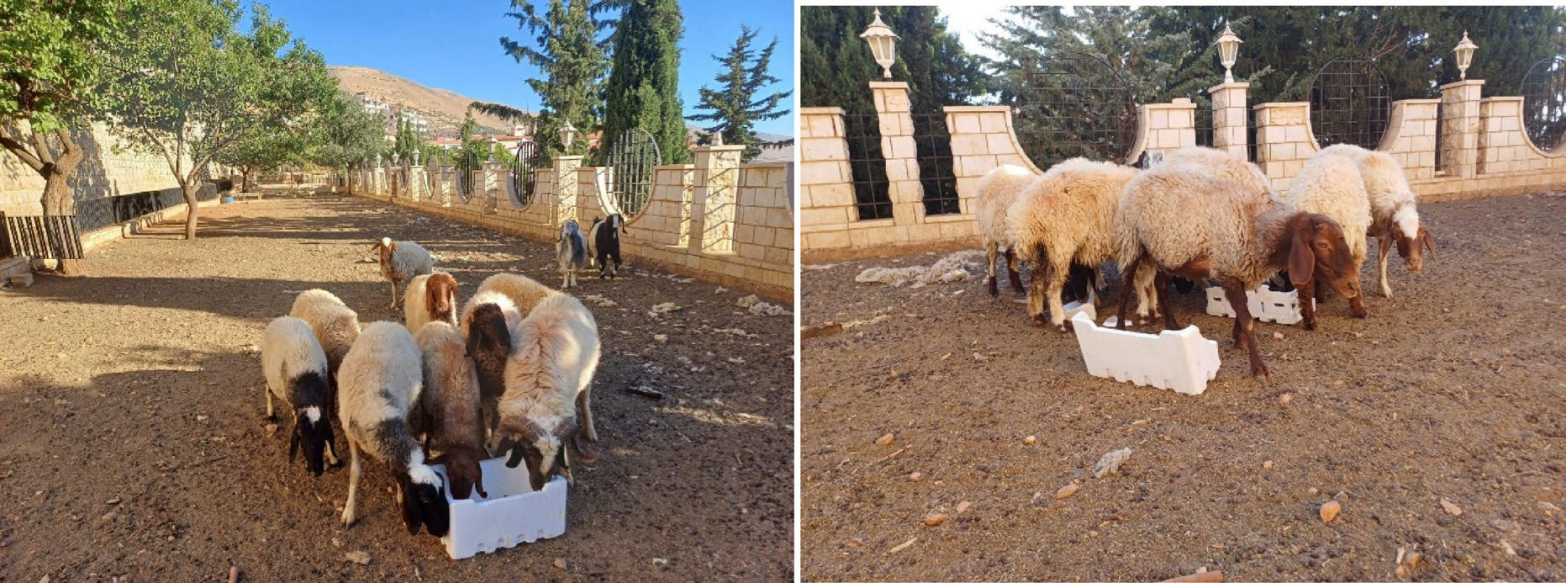
Sheep feeding near EPS-containing materials in a rural setting. The image illustrates the potential risk of accidental ingestion of expanded polystyrene (EPS) by grazing livestock, which may lead to digestive complications and broader implications for animal welfare and food safety.

Among the most concerning consequences of this dispersal is the accidental ingestion of EPS by grazing livestock such as sheep and goats. Field observations confirm that animals can mistake EPS fragments for forage, leading to gastrointestinal blockages, impaired digestion, and in severe cases, death. Figure 1 illustrates this risk, showing sheep feeding near EPS-containing materials in an agricultural setting. Recent physiological studies substantiate these observations: Chang et al. (2024) demonstrated that polystyrene exposure in lambs induces gastrointestinal injury, inflammation, and reduced growth and meat quality^3^. Furthermore, Aardema et al. (2024) emphasized that farm animals constitute a critical link between environmental microplastic pollution and human health, highlighting broader implications for food safety and agricultural productivity^4^.

In response to these environmental and agricultural concerns, this study aims to develop and evaluate a sustainable adhesive system derived from post-consumer EPS. By systematically investigating its chemical, rheological, and mechanical properties across multiple substrates, we seek to demonstrate the practical potential of EPS reuse while mitigating its ecological footprint.

Conventional disposal methods such as landfilling and incineration are constrained by spatial inefficiency and environmental impact. Dissolution-based recycling has thus emerged as a promising alternative, offering an energy-efficient and scalable route to polystyrene recovery. This approach involves solubilizing EPS in organic solvents to yield polymer gels or solutions that can be repurposed into adhesives, coatings, or composite materials^5^.

Previous research supports the feasibility of this strategy. García et al. (2009) examined polystyrene solubility and stability in various solvents, underscoring its potential for adhesive applications^6^. Selukar (2014) and Kaushal et al. (2018) explored EPS-derived adhesives for construction materials^7,8^, while De Paula et al. (2018) investigated its conversion into carbonaceous composites^9^. Notably, Uttaravalli et al. (2021) conducted a comprehensive study on EPS-based adhesives using six solvents—MEK, THF, n‑butyl acetate, m‑xylene, gasoline, and carbon tetrachloride—and reported that MEK-based formulations exhibited shear strength comparable to commercial adhesives like Fevicol, with successful bonding to wood, paper, and ceramic substrates^10^.

Building on this foundation, the present work introduces a novel adhesive system derived entirely from post‑consumer EPS, with a focus on mechanical performance across diverse substrates including wood, leather, ceramic, and polyurethane—the latter specifically relevant to footwear soles.

This study distinguishes itself by integrating rheological analysis, tensile testing, and substrate‑specific adhesion mechanisms, along with a comparative evaluation of solvent compatibility and additive effects. The inclusion of polyurethane extends the practical applicability of the adhesive, particularly in contexts such as shoe manufacturing and repair.

## Materials

### Expanded polystyrene (EPS) waste and bulk density determination

Post-consumer Expanded Polystyrene (EPS) waste was collected as the primary raw material. To accurately determine its characteristic low bulk density—a key factor in its environmental impact—a practical approach was employed. Multiple EPS packages were assembled to form a large composite structure with dimensions closely approximating 1 m³ (1 m × 1 m × 1 m). The total mass of this assembly was measured using a calibrated scale and found to be 1440 g. The bulk density was subsequently calculated as:

$$\rho=\frac{m}{V}=\frac{1440g}{1{m}^{3}}=1.440kg/{m}^{3}$$

**Fig. 2:**
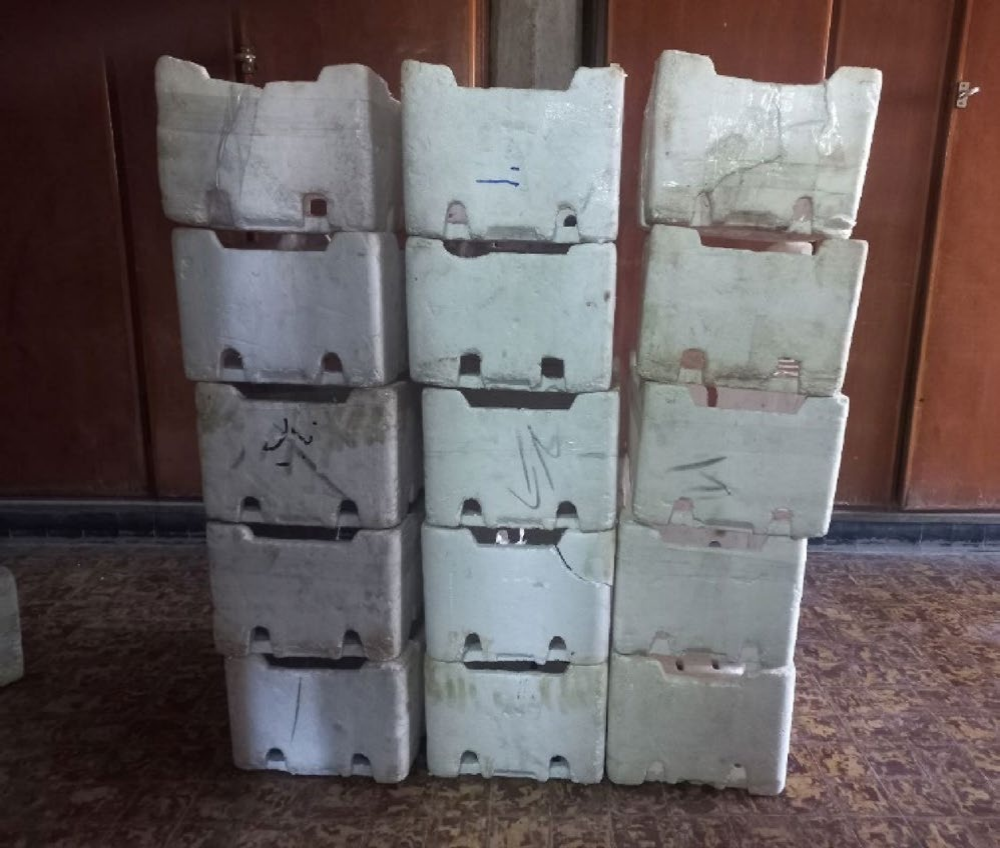
Photograph of the assembled EPS sample used for bulk density determination (approximate volume: 1 m³; measured mass: 1440 g).

This method effectively captures the real-world volumetric inefficiency of EPS waste. A photograph of the assembled sample is provided in Fig. 2 to illustrate the sampling geometry and scale.

### Solvents

The following organic solvents were used as received without further purification:

- **Methyl Ethyl Ketone (MEK)**, high purity (Puro), supplied by Carlo Erba (Code No. 354253).
- **Benzene**, 99.8% purity, supplied by Panreac (Code: 161192.1612).
- **Toluene**, PRS grade, supplied by Panreac (Code: 141745.1612).
- **Xylene**, supplied by Panreac, with a purity of 99.5% (by GC).

### Additive

Poly(methyl methacrylate) (PMMA) was supplied by Kumho Petrochemical Co., Ltd. (South Korea) and used as a reinforcing polymer additive.

## Methodology

### Sample preparation

Samples of recycled Expanded Polystyrene (EPS) were prepared by collecting EPS waste, shredding it into small pieces, and subsequently dissolving it in various organic solvents, including benzene, toluene, xylene, and methyl ethyl ketone (MEK). All basic adhesive formulations maintained a constant polymer-to-solvent mass ratio of 30:70, where the polymer component consisted solely of EPS.

Based on preliminary positive results in bonding applications on wood, two additional adhesive formulations were developed using methyl ethyl ketone as the primary solvent:

· The first formulation (EPS(M + T)) consisted of 30% EPS dissolved in a blended solvent system of 35% Methyl Ethyl Ketone and 35% Toluene.

· The second formulation (EPS(M+PMMA)) was modified by replacing part of the polymer content. It consisted of 30% EPS and 5% Poly (methyl methacrylate) (PMMA) as the polymer phase, dissolved in 65% Methyl Ethyl Ketone.

All mixtures were stirred using a mechanical stirrer at 100 RPM for 4 h at ambient temperature until complete dissolution was achieved. Table 1 details the complete compositions and nomenclature of all prepared samples.

### Chemical properties testing

#### Solubility determination

The solubility of EPS in the various solvents was determined gravimetrically, considering the minimum amount of solvent required to dissolve a pre-weighed sample of EPS (2 g). The solubility was calculated using the following Eq^6^. :

1 $$\mathrm{s}\mathrm{o}\mathrm{l}\mathrm{u}\mathrm{b}\mathrm{i}\mathrm{l}\mathrm{i}\mathrm{t}\mathrm{y}\left(\frac{\mathrm{g}}{\mathrm{m}\mathrm{l}}\right)=\frac{EPSweight\left(g\right)}{Solventvolume\left(ml\right)}$$

The experiment was repeated three times. The solubility values were consistent across all replicates, indicating good reproducibility. All measurements exhibited a coefficient of variation (CV) of less than 5.7%, confirming the accuracy and reliability of the obtained results.

**Table 1:** Composition and nomenclature of prepared EPS adhesive formulations.

dissolved substance	solvent	Additive	The symbol
Expanded Polystyrene (EPS)	Benzene		EPS(B)
	Toluene		EPS(T)
	Xylene		EPS(X)
	Methyl ethyl ketone		EPS(MEK)
	Toluene + Methyl ethyl ketone		EPS(T + MEK)
	Methyl ethyl ketone	PMMA	EPS (M+ PMMA)

#### Evaporation rate

The evaporation rate of the prepared adhesive samples was measured using a precise balance. The samples were weighed to four decimal places and their mass was measured again after one hour. The objective of this test was to determine the evaporation rate of the solvents in the EPS adhesives, to aid in the subsequent interpretation of adhesive strength. The evaporation rate was calculated as the percentage loss of mass (LM) using the following equation^11^:

2 $$LM=\frac{{m}_{i}-{m}_{f}}{{m}_{i}}\times100$$

Where ${m}_{i}$ and ${m}_{f}$ are the initial and final mass of the sample before and after solvent evaporation, respectively. All measurements showed high repeatability with a coefficient of variation (CV) of less than 0.11%, confirming the precision and reliability of the data.

### Rheological properties testing

The rheological properties of the samples were tested using a Fungilab Expert R Viscometer. To ensure accurate measurements, the spindle and rotational speed range were selected for each formulation to maintain the torque percentage within the valid instrument range of 20% to 90%. Accordingly, spindle R5 was used with a rotational speed range of 5, 7.5, 10, 12, 15, 20, 25, and 30 RPM for the EPS(B) sample. For the remaining formulations, spindle R4 was employed with a speed range of 35, 40, 50, 60, 70, 80, and 90 RPM.

#### Viscosity measurement and rheological analysis

The apparent viscosity of the prepared adhesive formulations was measured directly in centipoise (cP) using a Fungilab Expert R Viscometer. To enable comprehensive rheological characterization, the fundamental parameters of shear rate ($\dot{\gamma}$) and shear stress ($\tau$) were derived from the raw viscometer data using the following conversion equations:

3 $$\dot{\gamma}={K}_{1}\times{RPM}$$

4 $$\tau={K}_{2}\times\mathrm{T}\mathrm{o}\mathrm{r}\mathrm{q}\mathrm{u}\mathrm{e}\mathrm{\%}$$

Where K₁ and K₂ are factors dependent on the spindle number^12^.

K₁ = 0.21, 0.17 (sec⁻¹) for spindles R4 and R5, respectively.

K₂ = 0.430, 0.860 (dyne/cm²) for spindles R4 and R5, respectively.

#### Shear-thinning index (STI) test

The Shear-Thinning Index (STI), which serves as a tool for estimating the degree of non-Newtonian behavior, was determined using a method analogous to the ASTM E3070 standard. **(ASTM E3070-18 Standard Test Method**)^13^. This index is calculated from the difference in viscosity measured at the lowest and highest shear rates. An STI value of 0 indicates perfect Newtonian behavior, while an STI > 0 indicates shear-thinning (pseudoplastic) behavior. This index reflects the extent of polymer structural breakdown upon the application of shear and is also known as the Pseudoplastic Index. It was calculated as:

5 $$STI=\frac{{\eta}_{lowshear}-{\eta}_{haghshear}}{{\eta}_{lowshear}}$$

### Mechanical properties testing

**Fig. 3:**
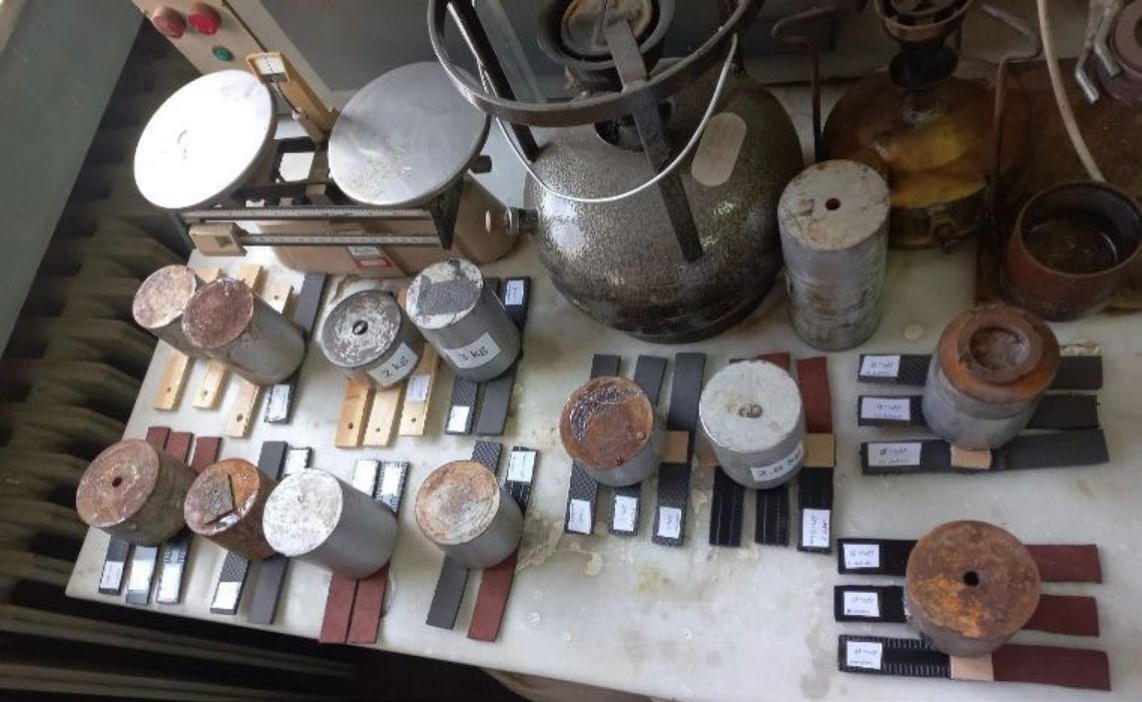
Labeled experimental samples prepared for tensile adhesion testing.

The adhesive strength (bonding strength) was evaluated via a tensile test using an Instron Universal Testing Machine. The adhesive was applied to substrates including leather, polyurethane foam, wood, and ceramic, with bonding dimensions of 25 mm × 25 mm and a thickness of 0.5 mm. As illustrated in Fig. 3, for each substrate type, 14 samples were prepared, and each sample was tested in triplicate to ensure statistical reliability. After application, a constant load was applied to each bonded specimen for 48 h to maintain uniform pressure and allow complete curing prior to testing. All tests were conducted at a controlled temperature of 22.8 °C and 50% relative humidity.

## Results and discussion

### Solubility determination

The solubility of EPS in various solvents was evaluated both experimentally and theoretically using the Hansen Solubility Parameters (HSP) framework. According to this model, the relative energy difference (RED), defined as RED = Ra/R₀, predicts solubility likelihood. An RED value less than 1 indicates a high probability of dissolution, while values increasingly greater than 1 suggest lower solubility^14^. The distance Ra between the solvent and polymer in Hansen space was calculated using the equation:

$$\left({R}_{a}\right)=\sqrt{4\varDelta{D}^{2}+\varDelta{P}^{2}+\varDelta{H}^{2}}$$

where ΔδD, ΔδP, and ΔδH are the differences in the dispersion, polar, and hydrogen-bonding solubility parameters, respectively, between the solvent and EPS^15^, and R₀ is the radius of the solubility sphere of EPS (12.7 MPa¹/²)^16^.

As presented in Table 2, the experimental solubility results show a strong correlation with the calculated RED values. Toluene, with the lowest RED value (0.65), exhibited the highest solubility (0.605 g/ml), whereas Methyl Ethyl Ketone, with the highest RED value (0.84), demonstrated the lowest solubility (0.487 g/ml). This remarkable agreement confirms the predictive power of the HSP model^17^. The superior performance of Toluene is attributed to its optimal combination of a low RED number, favorable molecular compatibility with non-polar EPS, and properties that facilitate rapid diffusion into the polymer’s cellular structure, as supported by its high self-diffusion coefficient reported in polystyrene solutions^18^. The intermediate solubility of Xylene (RED = 0.67, Solubility = 0.527 g/ml) and Benzene (RED = 0.67, Solubility = 0.495 g/ml) aligns with this rationale. The subtle performance difference between them can be attributed to their intrinsic physicochemical properties. Data from the DIPPR database indicates that Benzene possesses a higher volatility and a lower dynamic viscosity compared to Xylene. This likely causes Benzene to evaporate more rapidly during the dissolution process, thereby reducing its practical effectiveness despite its theoretical compatibility being nearly identical to that of Xylene^19^. In contrast, Methyl Ethyl Ketone’s higher polarity, reflected in its elevated RED value, along with its distinct solvent properties, fundamentally limit its interaction with the non-polar EPS matrix.

**Table 2:** Experimental Solubility of EPS and Correlation with Hansen Solubility Parameters.

Solvent	EPS solubility (g/ml)	$\boldsymbol{\delta}\boldsymbol{M}\boldsymbol{P}{\boldsymbol{a}}^{1/2}$			Vaper Pressure at 25 °C (KPa)	${\boldsymbol{R}}_{\boldsymbol{a}}$ (MPa ^0.5^)	$\boldsymbol{R}\boldsymbol{E}\boldsymbol{D}$ $={\boldsymbol{R}}_{\boldsymbol{a}}/{\boldsymbol{R}}_{0}$
		${\boldsymbol{\delta}}_{\boldsymbol{D}}$	${\boldsymbol{\delta}}_{\boldsymbol{P}}$	${\boldsymbol{\delta}}_{\boldsymbol{H}}$			
EPS	-	21.3	5.8	4.3	-	-	-
Toluene	0.605 $\pm$ 0.053	18	1.4	2	3.87	8.26	0.650
xylene	0.527 $\pm$ 0.027	17.8	1	4.1	1.17	8.49	0.668
Benzene	0.495 $\pm$ 0.001	18.4	0	2	12.58	8.52	0.670
Methyl ethyl Ketone	0.487$\pm$ 0.001	16	6.3	5.1	10.37	10.64	0.837

*Note: The Hansen parameters for EPS and solvent are from reference data **(Burke**,** J. (1984))**^20^, (**Lide**,** D. R. (1991))**^21^. *.

### Evaporation rate

As delineated in Table 3, the evaporation rates of the prepared adhesive formulations exhibit a clear correlation with the intrinsic volatility of their constituent solvents. The EPS(B) sample demonstrated the highest evaporation rate (35.19%), which is consistent with the high volatility inherent to benzene. This was followed by EPS(X), EPS(T), and the blend EPS(M + T). A significant decrease in evaporation rate was observed for the MEK-based formulations, with EPS(MEK) and EPS(M+PMMA) showing the lowest values (28.45% and 24.87%, respectively). This can be attributed to the lower volatility of MEK and the further vapor-pressure depression caused by the dissolution of the PMMA polymer additive.

**Table 3:** Evaporation rates of the prepared EPS adhesive formulations.

Samples	Evaporation rate (%)
EPS(T)	34.13$\pm$ 1.45
EPS(X)	34.31$\pm$ 2.52
EPS(B)	35.19$\pm$ 0.40
EPS(MEK)	28.45$\pm$ 1.99
EPS (M + T)	33.74$\pm$2.68
EPS (M+ PMMA)	24.87$\pm$ 1.88

An important experimental observation for the MEK-based adhesives was the rapid formation of a superficial skin or layer at the air-adhesive interface immediately after application. This phenomenon, where solvent evaporation leads to a concentrated polymer layer that can hinder further solvent loss from the underlying bulk, is a recognized characteristic in polymer solutions and adhesive science^11^. This skin formation effectively reduces the overall evaporation rate and can influence the adhesive’s open time and bonding characteristics.

### Rheological properties

**Fig. 4:**
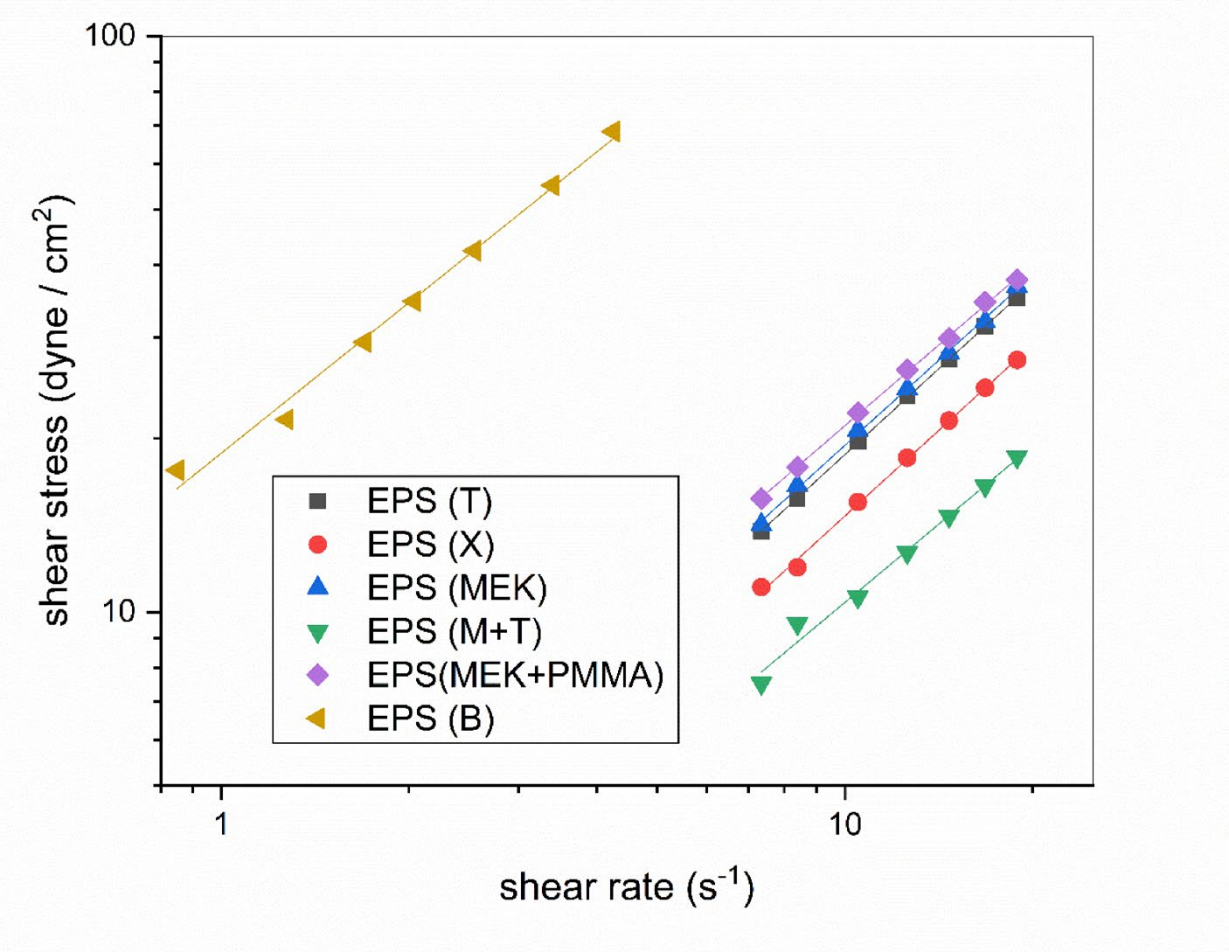
Flow curves of the prepared EPS adhesive formulations.

The rheological behavior of the prepared adhesive formulations was analyzed to assess their flow characteristics, which are critical for application performance. Figure 4 illustrates the relationship between shear stress and shear rate for all samples, fitted using the Power-Law model (τ = Kγⁿ). As summarized in Table 4, the consistency index K showed significant variation among samples, with EPS(B) exhibiting the highest value (18.87 dyne·sⁿ/cm²), indicating greater resistance to flow, while other formulations showed substantially lower values ranging from 1.29 to 2.46 dyne·sⁿ/cm².

The flow behavior indices (n) ranged between 0.868 and 0.989, indicating near-Newtonian to weakly shear-thinning behavior. The high n values for EPS(T) and EPS(X) (0.989 and 0.986, respectively) confirm their approximately Newtonian flow, consistent with their excellent solvent quality. This near-Newtonian behavior in well-compatible polymer-solvent systems has been previously reported by (Serin et al., 2016). for polystyrene solutions^22^.

The Shear-Thinning Index (STI) provides further insight into the non-Newtonian character. The STI values, calculated according to Eq. (5), ranged from 0.018 for EPS(X) to 0.203 for EPS(B). The low STI values for EPS(T) and EPS(X) confirm their nearly Newtonian flow, attributed to their high compatibility with EPS, as indicated by their low RED values, which promotes effective chain dissolution and minimal structural resistance to shear. In contrast, EPS(B) exhibited the highest STI value, signifying the most pronounced shear-thinning behavior^23^. This can be linked to its higher consistency index (K) and the use of a different spindle geometry, which may have induced localized shear deformations. The moderate STI values for MEK-based systems (EPS(MEK) and EPS(M+PMMA)) suggest that despite the solvent’s higher polarity, these formulations still display measurable non-Newtonian character, potentially due to dipole-dipole interactions promoting temporary clustering.

**Table 4:** Rheological parameters of EPS adhesive formulations obtained from Power-Law model fitting and Shear-Thinning Index (STI).

Samples	EPS (T)	EPS (X)	EPS (B)	EPS (MEK)	EPS (M + T)	EPS (M+ PMMA)
n	0.989	0.986	0.868	0.981	0.902	0.931
K (dyne.s^n^/cm^2^)	1.92	1.51	18.87	2.03	1.29	2.46
R^2^	0.999	0.997	0.991	0.998	0.987	0.999
STI	0.035	0.018	0.203	0.052	0.027	0.066

**Fig. 5:**
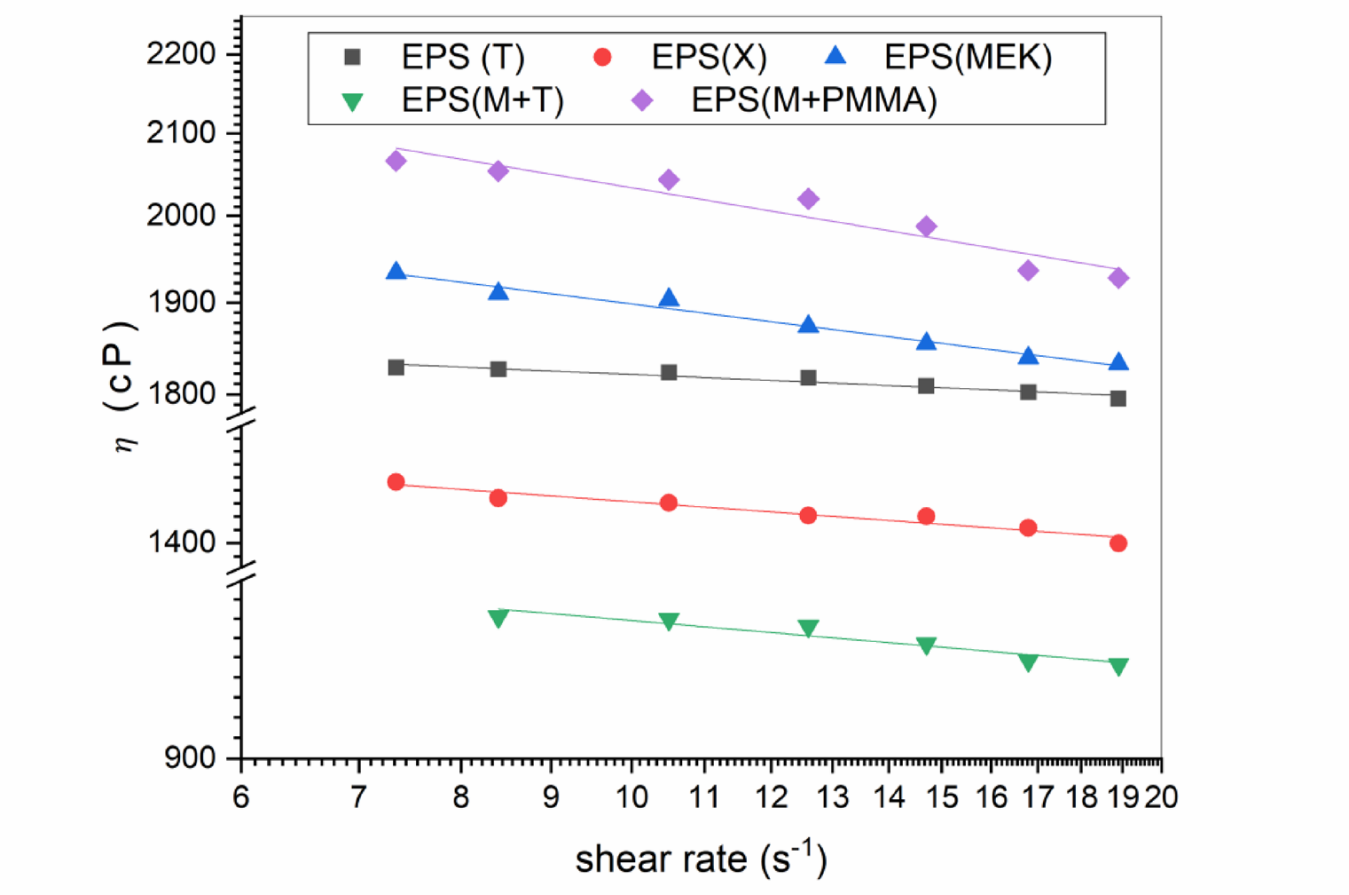
Apparent viscosity as a function of shear rate for the EPS adhesive formulations.

The viscosity profiles, depicted in Fig. 5, show a slight shear-thinning tendency, particularly for the EPS(MEK) system. The EPS (MEK) and EPS (B) combinations showed a slight downward slope, consistent with their higher STI values. Conversely, EPS(T) and EPS(X) maintained almost constant viscosity across the measured shear rate range, underscoring their Newtonian-like response and excellent solvent quality, which reduces viscosity dependence on shear.

From a processing standpoint, these results highlight how solvent selection governs adhesive behavior during application. Systems with nearly constant viscosity (X and T) are ideal for controlling spread and film uniformity.as discussed in rheological studies of adhesive systems^24^. In contrast, formulations with higher K and moderate shear-thinning (B and MEK) may offer advantages like enhanced wetting under high shear and improved cohesion at rest, a rheological duality crucial for optimizing adhesive performance.

### Mechanical properties

#### Performance on leather substrate

**Fig. 6:**
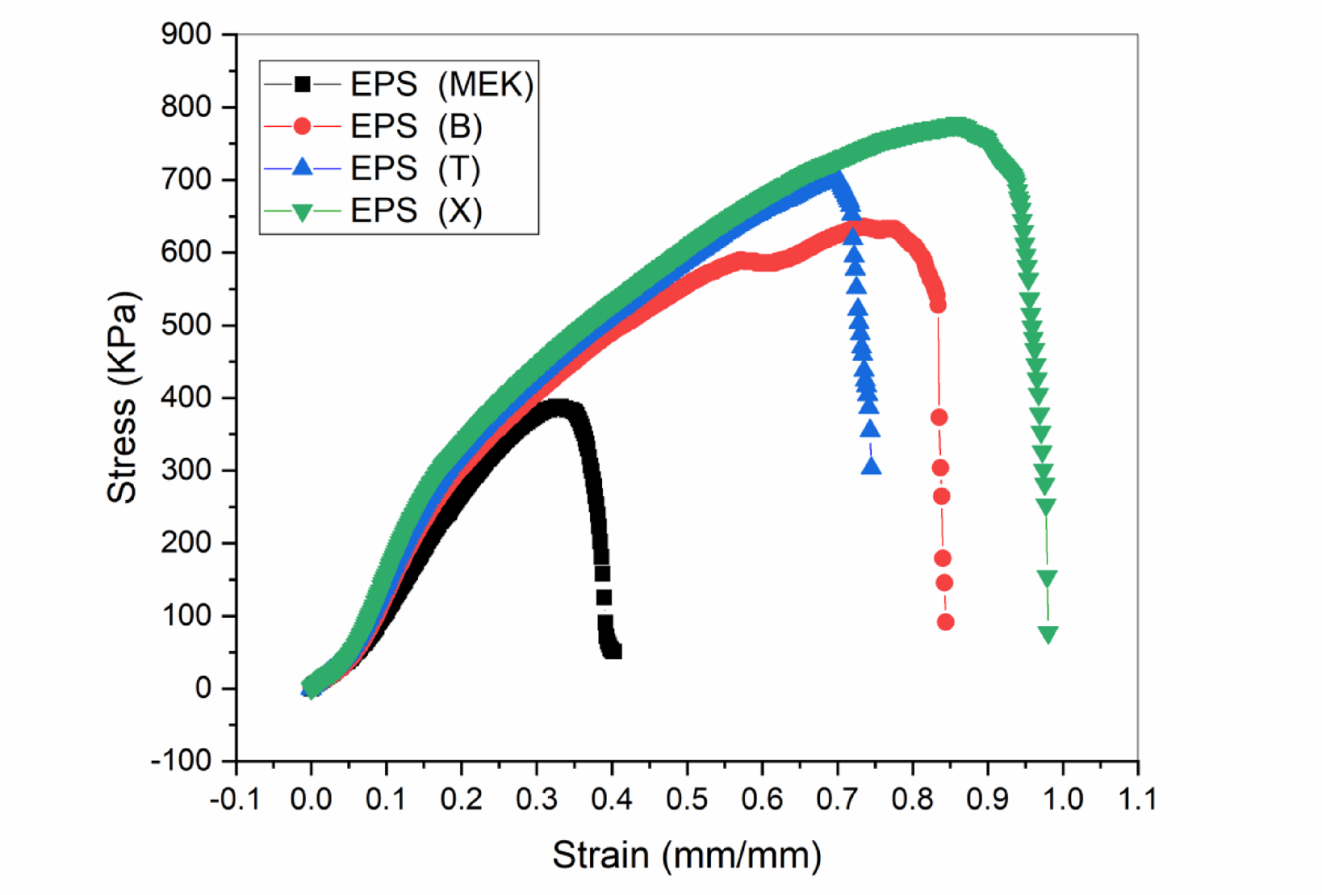
Tensile stress-strain curves of the prepared EPS adhesive formulations on leather substrate.

Table 5; Fig. 6 shows that sample EPS(X) recorded the highest maximum stress on belt leather, reaching 778.069 KPa, outperforming EPS(T) (700.39 KPa) and EPS(B) (632.4 KPa). This superiority is explained by the balanced interplay between the adhesive’s rheological and chemical properties.

The superior performance of EPS(X) on leather (778.069 KPa) is attributed to a synergistic combination of favorable rheology and chemical compatibility. Its near-Newtonian behavior (*n* = 0.986) ensures uniform application and stable viscosity under shear, preventing premature structural collapse during spreading. Chemically, the low RED value (0.668) indicates high affinity between xylene and the non-polar EPS matrix, promoting efficient dissolution and chain mobility. This, coupled with an optimal evaporation rate (34.31%), facilitates deep penetration into the leather’s micro-porous structure, forming robust mechanical interlocks. In contrast, the pronounced shear-thinning of EPS(B) (STI = 0.203) and its higher volatility likely hinder penetration, resulting in weaker interfacial bonding.

Consequently, EPS(X)’s advantage over toluene and benzene formulations stems from an optimal balance of viscosity, evaporation rate, and chemical compatibility, making it superior on belt leather in terms of adhesive strength and application stability. By contrast, EPS(MEK) exhibited the lowest maximum stress (306.97 KPa), associated with its low evaporation rate (23.45%) and higher RED (0.837), indicating poor compatibility with the base polymer, reduced penetration into the leather, and lower bond strength.This observed trend aligns with the diffusion theory of adhesion, where effective polymer chain interlocking is governed by solvent quality and evaporation kinetics^11,25^.

#### Performance on wood substrate and performance enhancement with additives

**Fig. 7:**
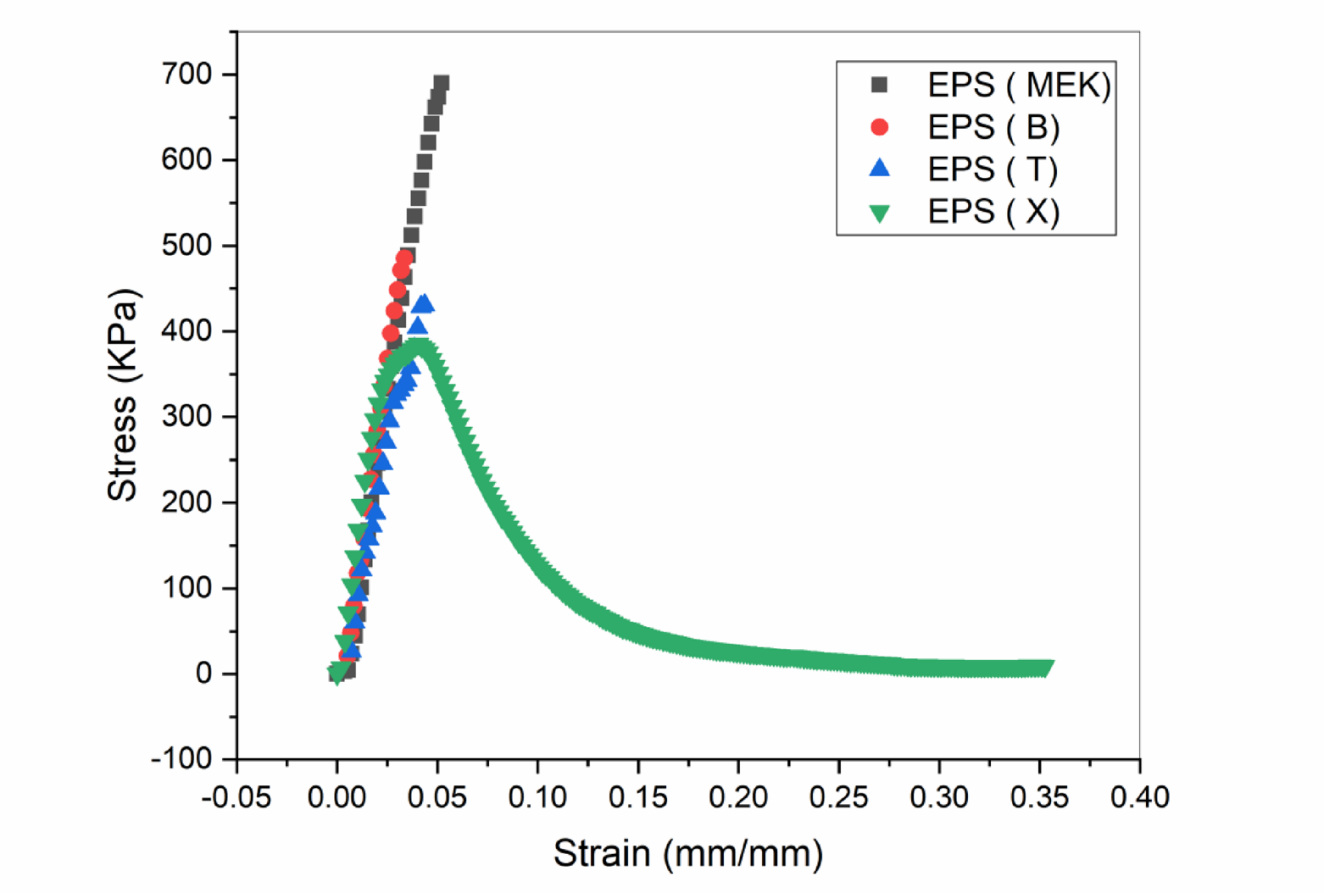
Tensile stress-strain curves of the prepared EPS adhesive formulations on wood substrate.

The mechanical performance of EPS-based adhesives on wood varied significantly across formulations (Fig. 7; Table 5). EPS(MEK) exhibited the highest tensile strength (754.78KPa), followed by EPS(B) (707.58 KPa) and EPS(T) (604.72 KPa). In contrast, EPS(X) showed a substantially lower strength (445.49 KPa) and no measurable spontaneous breaking stress. This limited performance is likely due to its rapid evaporation and quasi-Newtonian behavior (*n* = 0.986, STI = 0.018), which favors surface-level coverage over deep penetration into the porous wood matrix, potentially resulting in adhesive or interfacial failure during testing.

The superior adhesion of EPS(MEK) stems not only from its moderate evaporation rate (28.45%) but also from its intrinsic chemical affinity for wood. As a polar aprotic solvent, methyl ethyl ketone (MEK) contains a carbonyl group capable of forming hydrogen bonds with the hydroxyl-rich cellulose in wood. These interactions enhance interfacial adhesion via dipole–dipole and hydrogen-bonding mechanisms, thereby improving mechanical stability and bond strength. This aligns with the diffusion theory of adhesion, which underscores the importance of molecular interactions and solvent–substrate compatibility^25–28^. Recent reviews further emphasize how solvent polarity and functional group availability modulate hydrogen bonding in wood adhesion^26,29^, and studies confirm that ketone-based solvents such as MEK can engage in transient hydrogen bonding with cellulose, influencing both wetting and adhesion^30,31^.

Performance was further elevated with the addition of poly (methyl methacrylate) (PMMA) in the EPS(M+PMMA) formulation, which achieved a remarkable tensile strength of (1968.62KPa). Beyond simply reinforcing the polymer matrix, PMMA introduces additional polar carbonyl groups that can form hydrogen bonds with cellulose hydroxyls, strengthening interfacial compatibility and molecular anchoring^26,32,33^. PMMA and its copolymers are known to establish stable hydrogen-bonded networks with cellulose derivatives, affecting both adhesion and viscoelastic behavior at the interphase^29,30^. For instance, Fahrländer et al. (2001) showed that PMMA particles alter the rheology of polystyrene matrices, enhancing structural cohesion and flow characteristics via interfacial interactions and phase morphology^34^. Thus, PMMA acts synergistically as both a mechanical reinforcer and a chemical mediator that promotes interfacial cohesion and rheological stability.

**Table 5:** Caption: Mechanical performance of EPS adhesive samples on substrates (maximum stress and displacement data).

Substrate	Leather				
Samples	STRESS (Max Load) (KPa)	Strain (Max Disploment) (mm/mm)	STRESS (Load Auto Break) (KPa)		Strain (Disploment at Auto Break) (mm/mm)
EPS (T)	700.39	0.694	577.34		0.72
EPS (X)	778.069	0.857	629.34		0.947
EPS (B)	632.4	0.760	527.89		0.833
EPS (MEK)	306.97	0.371	158.14		0.387
Substrate	Polyurethane Foam				
	The sample was cut outside the adhesion area.				
Substrate	Wood				
EPS (T)	604.72	0.0372		1841.21	0.0359
EPS (X)	445.49	0.1255		No value	No value
EPS (B)	707.58	0.0275		1543.97	0.0258
EPS (MEK)	754.78	0.0481		3184.44	0.0471
EPS (M + T)	135.250.	0.0323		1081.48	0.028
EPS (M+PMMA)	1968.62	0.0295		2010.88	0.0284

Additional benefits of PMMA include improved film integrity, reduced solvent evaporation, and more uniform stress distribution—all critical for durable adhesion^27^. The enhanced performance of EPS(M+PMMA) is therefore attributed to a dual mechanism: (1) mechanical reinforcement of the adhesive matrix and (2) interfacial synergy through hydrogen bonding and polarity matching with cellulose^26,35^.

Conversely, the EPS(M + T) formulation displayed markedly lower strength (135.25 KPa), likely due to competitive solvent interactions that disrupt optimal hydrogen bonding and reduce permeability. These results underscore the critical influence of solvent–substrate compatibility and molecular-level interactions on adhesive performance.

#### Performance on polyurethane substrate

Tensile testing on polyurethane foam—representative of commercial shoe sole material—revealed a consistent and significant failure mode across all adhesive formulations. In every case, rupture occurred cohesively within the polyurethane substrate itself, rather than at the adhesive–substrate interface or within the adhesive layer. This cohesive substrate failure provides definitive evidence that the adhesive strength of every EPS-based formulation surpassed the intrinsic cohesive strength of the polyurethane material. Such a failure mode represents an ideal bonding outcome in practical terms, particularly for footwear repair and manufacturing, where adhesion must reliably exceed the substrate’s own integrity.

While this failure mode precluded the extraction of precise numerical adhesive strength values, the consistent observation of substrate failure unequivocally validates the exceptional bonding capability of the developed adhesives for shoe-sole applications. This interpretation aligns with established adhesive testing standards (e.g., ASTM D4541/D7234), where substrate failure is recognized as an indicator of optimal adhesive performance^29^.

#### Performance on ceramic substrate

**Fig. 8:**
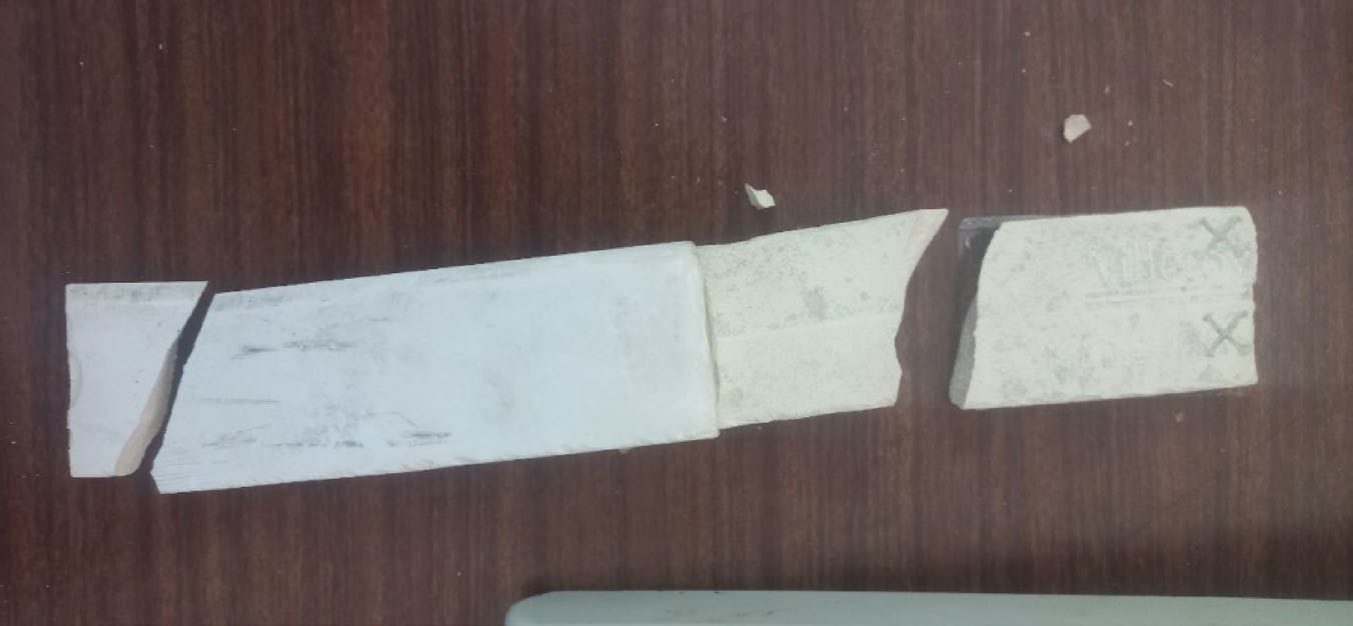
Fracture of ceramic specimens during tensile testing due to brittle failure at the gripping jaws.

The intrinsically brittle nature of ceramics presented significant challenges for conventional tensile testing. As shown in Fig. 8, ceramic specimens consistently underwent premature fracture at the gripping points under minimal applied stress, precluding the collection of reliable quantitative tensile data. This behavior aligns with the well-documented low fracture toughness of ceramic materials, which renders them susceptible to catastrophic failure under tensile loading.

**Fig. 9:**
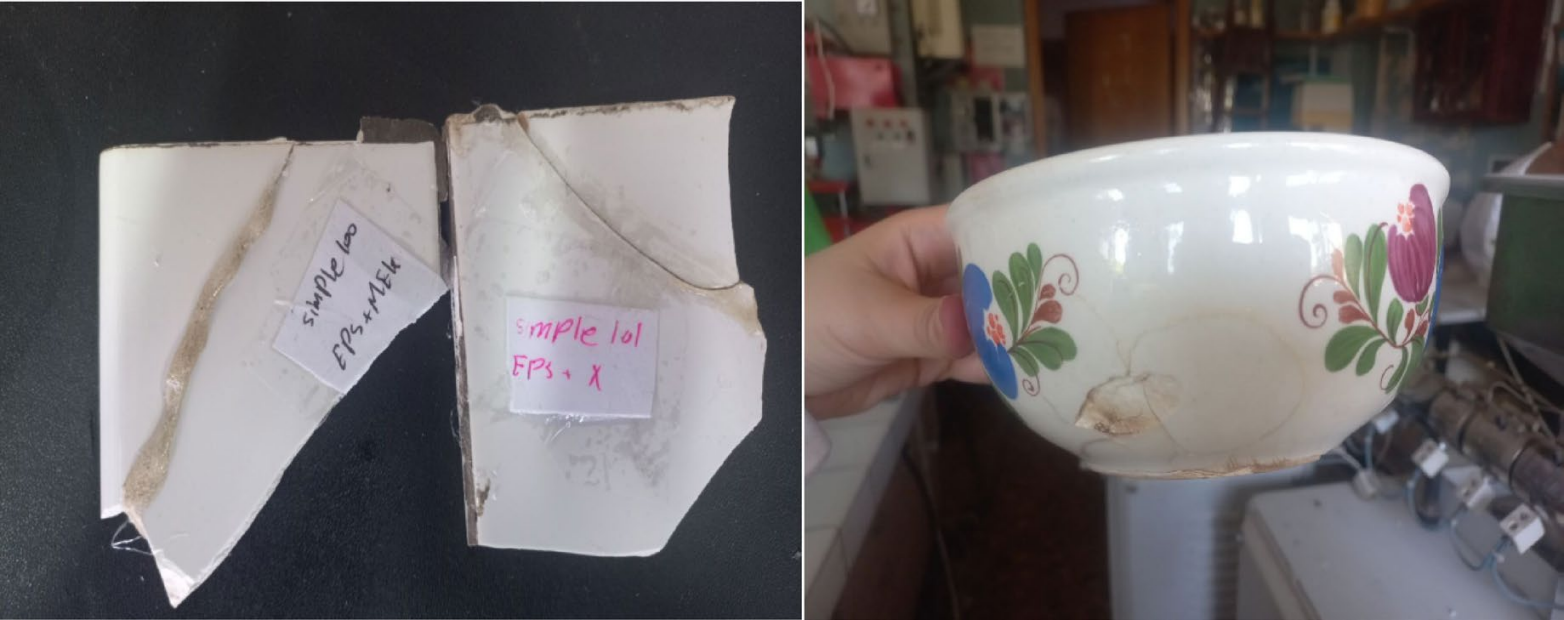
Adhesive application on ceramic substrate showing intact bonding interface after manual tensile loading.

Despite these limitations, the adhesive’s performance on ceramic substrates was effectively assessed through qualitative pull-off tests. Following application and curing, the bonded ceramic interface demonstrated remarkable mechanical integrity, resisting manual separation attempts (Fig. 9). This robust adhesion, even under qualitative hand-driven loading^10^, indicates strong interfacial compatibility. The observed performance is likely attributable to effective adhesive wetting and micro-mechanical interlocking with the textured ceramic surface, compensating for the lack of quantitative tensile metrics.

### Sustainability considerations and future directions

While the present study validates the technical feasibility of recycling EPS waste into high‑performance adhesives, broader sustainability considerations must be acknowledged. The valorization of post‑consumer EPS contributes to reducing plastic waste volume and landfill burden, thereby supporting circular‑economy principles^35^. However, the solvents employed—benzene, toluene, xylene, and MEK—are associated with environmental and health risks. Benzene is classified as a human carcinogen, while toluene and xylene are known to cause neurological effects upon prolonged exposure. MEK, although less toxic, can still induce irritation and requires controlled handling^36,37^. Thus, strict safety protocols and the exploration of greener, bio‑based alternatives are essential to align adhesive development with sustainability goals^38^.

Although the adhesive developed in this study is not biobased in its chemical origin, its environmental relevance aligns with the broader principles of biobased and low‑impact materials. Recent literature highlights those sustainable adhesive technologies are not limited to biomass‑derived polymers but also include systems that reduce fossil‑resource consumption and overall carbon footprint through recycling and circular‑economy strategies. Upcycling post‑consumer EPS into a functional adhesive directly offsets the production of virgin polystyrene and reduces landfill accumulation, thereby lowering greenhouse‑gas emissions associated with polymer manufacturing. This perspective is consistent with modern sustainability frameworks, which classify waste‑derived materials as contributors to carbon reduction and resource efficiency, similar to the environmental benefits reported for biobased wood adhesives in recent studies such as Calvez et al. (2024), Calovi et al. (2024), and Ghaffar & Fan (2024, Coatings)^39–41^. Therefore, the recycled EPS adhesive developed here provides an environmentally favorable alternative to conventional solvent‑borne systems, even if not biobased in origin.

In addition, although the mechanical data presented here provide strong evidence of substrate‑specific performance, direct characterization of the adhesive bonding mechanism remains limited. Future studies should employ contact angle measurements to quantify wetting behavior and FTIR spectroscopy to probe hydrogen bonding and interfacial chemistry, thereby offering more definitive mechanistic insights^25^. Furthermore, comparative evaluation between adhesives derived from recycled EPS and those prepared from virgin PS would clarify the performance trade‑offs and reinforce the sustainability justification of recycling^25,38^.

By integrating these considerations, subsequent research can strengthen both the environmental relevance and the scientific rigor of EPS‑based adhesives, ensuring that their application not only addresses waste‑management challenges but also advances sustainable materials science.

## Conclusions

Collectively, this work establishes a sustainable pathway for valorizing EPS waste into high-performance adhesives, underpinned by a solvent-substrate design principle. By strategically matching solvent polarity with substrate chemistry—non-polar aromatics for leather, polar ketones for wood—we demonstrate that tailored formulations can compete with conventional adhesives. This approach not only mitigates plastic pollution but also offers a versatile material solution for industries ranging from footwear to construction, highlighting the potential of waste-centric circular economy models.

## Data Availability

All data generated or analyzed during this study are included in this published article.
